# Sequence Variation in Caprine *KRTAP6-2* Affects Cashmere Fiber Diameter

**DOI:** 10.3390/ani12162040

**Published:** 2022-08-11

**Authors:** Jian Cao, Jiqing Wang, Huitong Zhou, Jiang Hu, Xiu Liu, Shaobin Li, Yuzhu Luo, Jon G. H. Hickford

**Affiliations:** 1 Gansu Key Laboratory of Herbivorous Animal Biotechnology, Faculty of Animal Science and Technology, Gansu Agricultural University, Lanzhou 730070, China; 2 Gene-Marker Laboratory, Department of Agricultural Sciences, Lincoln University, Lincoln 7647, New Zealand

**Keywords:** keratin-associated protein 6-2 (KAP6-2), *KRTAP6-2*, variation, mean fiber diameter (MFD), cashmere, goat

## Abstract

**Simple Summary:**

Keratin-associated proteins (KAPs) are a structural component of wool fibers. Despite the gene encoding the high-glycine/tyrosine KAP6-2 (called *KRTAP6-2*) having been described in sheep, it has not been identified goats. This study describes the identification of caprine *KRTAP6-2*, reports variation in the gene, and describes its association with cashmere fiber diameter in Longdong cashmere goats.

**Abstract:**

Keratin-associated proteins (KAPs) are a structural component of cashmere fibers and in part determine fiber attributes. The gene encoding the high-glycine/tyrosine KAP6-2 (called *KRTAP6-2*) has been described in sheep, but it has not been identified goats. In this study, a 252-bp open reading frame with similarity to ovine *KRTAP6-2* was found on goat chromosome 1, with its upstream and downstream flanking sequences are closely related with ovine *KRTAP6-2* but are clearly distinct from other ovine *KRTAP6-n* sequences. Polymerase chain reaction amplification followed by single strand conformation polymorphism analysis of this region revealed five distinct banding patterns representing five different sequences (*A* to *E*) in 230 Longdong cashmere goats. Eleven diallelic single nucleotide polymorphisms (SNPs), a three-nucleotide sequence variation, and a 12-bp insertion/deletion were found among these five sequences, with most SNPs being either outside the coding region or synonymous. The presence of variant *D* was found to be associated with decreased mean fiber diameter (MFD; present: 13.26 ± 0.07 µm; absent: 13.55 ± 0.04 µm; *p* < 0.001), suggesting that variation in *KRTAP6-2* may affect fiber diameter and have value as a molecular marker for improving the cashmere fiber diameter trait.

## 1. Introduction

Cashmere fibers are non-medullated and produced by the secondary follicles in cashmere goat skin. They are used to manufacture luxury textile products due to their softness, lightness, and warmth. The yield of cashmere fiber, the mean fiber diameter (MFD), and fiber length are important traits as they determine the economic return for cashmere production [1]. These are moderate to highly heritable traits [2]. As the quantity and quality of cashmere fibers are affected by environmental, genetic, and nutritional influences, the identification of genes that affect cashmere fiber traits provides one basis for fiber improvement.

The fibers from cashmere goats contain keratin (K) proteins and keratin-associated proteins (KAPs), with the Ks assembled into keratin intermediate filaments (KIFs), while KAPs form a milieu that cross-links the KIFs [3]. Consequently, the Ks and KAPs have a role in defining the properties of fibers. 

The KAPs are proteins that range from 10–30 kDa in size and usually have either a high content of the amino acid cysteine, or glycine and tyrosine [3,4]. They have been categorized into three groups: high-glycine/tyrosine KAPs (35–60 mol % glycine and tyrosine), high-sulfur KAPs (≤30 mol % cysteine), and ultra-high-sulfur KAPS (>30 mol % cysteine) [5].

The KAPs are encoded by small intron-less genes called *KRTAPs* [5]. While 88 functional *KRTAPs* from 25 families have been identified in humans, and 30 *KRTAPs* comprised of 18 families have been reported in sheep, only 18 *KRTAPs* and 12 families having been identified in goats [6]. Of the 18 caprine *KRTAPs* identified, variation in 10 of these genes has been reported to be associated with selected cashmere fiber traits, including *KRTAP1-3* [7], *KRTAP8-2* [8], *KRTAP9-2* [9], *KRTAP13-1* [10], *KRTAP15-1* [11], *KRTAP20-1* [12], *KRTAP20-2* [1], *KRTAP24-1* [13], *KRTAP27-1* [14], and *KRTAP28-1* [15].

Previous studies have identified HGT-*KRTAPs* that are present in sheep and goats but are absent in humans [8,16,17,18], with this suggesting a difference in the complexity of HGT-KAPs between wool/cashmere fibers and human hair. Of all the HGT-*KRTAPs* identified for sheep, the highest level of heterogeneity is observed for the KAP6 family, for which five members are present in sheep compared to three in humans [17,19].

No *KRTAPs* in the KAP6 family have been identified in goats. Despite there being two goat *KRTAP* sequences (EU145019 and AY316158; [20,21]) reported to be “alleles of caprine *KRTAP6-2*”, these two sequences do not share high similarity to any *KRTAP6-n* sequences from sheep or humans [6]. This suggests that the reported sequences may not be from the caprine *KRTAP6-2*, and that caprine *KRTAP6-2* remains to be identified.

In this research, we aimed to find the KAP gene *KRTAP6-2* in cashmere producing Longdong goats. We used a polymerase chain reaction-single strand conformation polymorphism (PCR-SSCP) analysis to distinguish nucleotide sequence differences in the gene, and to investigate whether these differences were predictive of variation in selected important cashmere fiber traits.

## 2. Materials and Methods

### 2.1. Description of the Longdong Casgmere Goats Investigated, and Sample and Data Collection

Longdong cashmere goats (*n* = 230 in total) were studied. These were raised in the Gansu Province of China by the Yusheng Cashmere Goat Breeding Company. When the goats were approximately one year old, their fleece was combed to retrieve the cashmere fiber, as is the traditional practice. The weight of fiber retrieved for each goat was recorded. A small sample of fiber was specifically collected from each goat’s mid-side region to enable the determination of the mean fiber diameter (MFD) and fiber length using the Optical Fiber Length and Diameter Analyzer (OFDA4000; EPCO, Shanghai, China). At the time of fiber collection, a small sample of blood was retrieved from the ear of each goat and absorbed onto separate FTA^TM^ cards (Whatman BioScience, Middlesex, UK). The samples were air-dried and stored in the dark at room temperature until required for further analysis. Genomic DNA that binds to the FTA^TM^ was prepared for PCR amplification using an approach defined by Zhou et al. [22]. Briefly, a 1.2 mm disk was excised from the blood sample on the card and put in a 0.7-mL tube containing 200 µL of 20 mM NaOH. These samples were incubated for 20 to 30 min at 60 °C. The liquid was then aspirated, and the disk equilibrated in 200 µL of TE^−1^ buffer (10 mM Tris-HCl, 0.1 mM EDTA, pH 8.0). The buffer was then removed, and the disks allowed to air dry.

### 2.2. PCR-SSCP Analysis of Caprine KRTAP6-2

To identify caprine *KRTAP6-2,* an ovine gene sequence (accession number KT725827) was used to search the caprine ASM170441v1 genome assembly. The sequence that shared the most similarity to ovine *KRTAP6-2* was expected to be caprine *KRTAP6-2*. Based on this caprine sequence, PCR primers (5′-GAGAAATGTCCACACTCAAGT-3′ and 5′-GAGGGCATTAAAAGGCACGT-3′; synthesized by the Takara Biotechnology Co., Ltd., Dalian, China) were designed to amplify a 430-bp portion of the caprine DNA that encompassed the entire coding region of what was presumed to be the caprine *KRTAP6-2*.

Sequence amplification with PCR was undertaken in a 20-µL reaction comprising the genomic DNA on the 1.2-mm punch of blood, 0.25 µM of each primer, 150 µM of the four dNTPs (Takara, Dalian, China), 2.5 mM Mg^2^^+^, 0.5U of Taq DNA polymerase (Takara, Dalian, China), and 1× PCR reaction buffer that was supplied with the polymerase enzyme. The thermal cycling procedure included incubation for 2 min at 94 °C, followed by 35 cycles of 94 °C for 30 s, 60 °C for 30 s, and 72 °C for 30 s. A final extension of 5 min at 72 °C was used to ‘polish’ any incomplete amplicons. Thermal cycling was carried out in Bio-Rad S1000 thermal cyclers (Bio-Rad, Hercules, CA, USA).

Upon completion of the amplifications, the amplicons were examined using a SSCP method. For these analyses, a 1-µL aliquot of the amplicon was combined with 7 µL of loading dye (98% formamide, 10 mM EDTA, 0.025% bromophenol blue and 0.025% xylene-cyanol). These samples were incubated at 95 °C for 10 min to denature the DNA to a single-strand state, then quickly cooled on wet ice. They were immediately loaded onto 16 × 18 cm, 12% acrylamide: bisacrylamide (37.5:1) (Bio-Rad) gels that contained 0.2% *v*/*v* glycerol. Electrophoresis was performed in 0.5× TBE buffer at 230 volts and 18 °C for 21 h. A method described by Byun et al. [23] was used to stain the gels and reveal band patterns.

### 2.3. DNA Sequencing and Sequence Analyses 

Following the PCR-SSCP analysis, amplicons that were observed to be homozygous were subjected to direct DNA sequence using the original PCR primers. However, for amplicons that were only found in a heterozygous form, the DNA templates for subsequent sequencing were produced using an approach that has been described by Gong et al. [24]. All the DNA sequencing was conducted at the Beijing Genomics Institute (Beijing, China).

The online DNA sequence analysis tool Open Reading Frame Finder (https://www.ncbi.nlm.nih.gov/orffinder/) was used to find any open reading frames in the DNA sequences that were produced. DNAMAN (version 5.2.10 Lynnon BioSoft, Vaudreuil, Canada) was then used to align and compare DNA sequences, and to construct phylogenetic trees. The BLAST algorithm (http://blast.ncbi.nlm.nih.gov/; accessed on 21 May 2022) was used to search the NCBI GenBank databases for sequences homologous to those obtained from the amplicons. 

### 2.4. Statistical Analyses 

All data were analyzed using SPSS v24.0 (IBM, Armonk, NY, USA). General linear mixed models (GLMMs) were used to evaluate associations between the presence or absence of individual variants of caprine *KRTAP6-2* and variation in the crimped fiber length, cashmere yield and MFD. Gender and sire were revealed in ANOVAs to affect all the fiber traits (*p* < 0.05), so they were fitted as fixed and random factors, respectively. Birth rank did not affect fiber traits (*p* > 0.05), so it was not included as a factor in the models.

## 3. Results

### 3.1. Identification of Caprine KRTAP6-2 

A BLAST search of the Caprine Genome Assembly NC_030808.1 using an ovine *KRTAP6-2* sequence (KT725827.1) revealed a highly similar region (identity 97%, E value 0.0) on chromosome 1. A 252-bp open reading frame (ORF) was found within this region at the location NC_030808.1: nt3536200-nt3536451.

**Figure 1:**
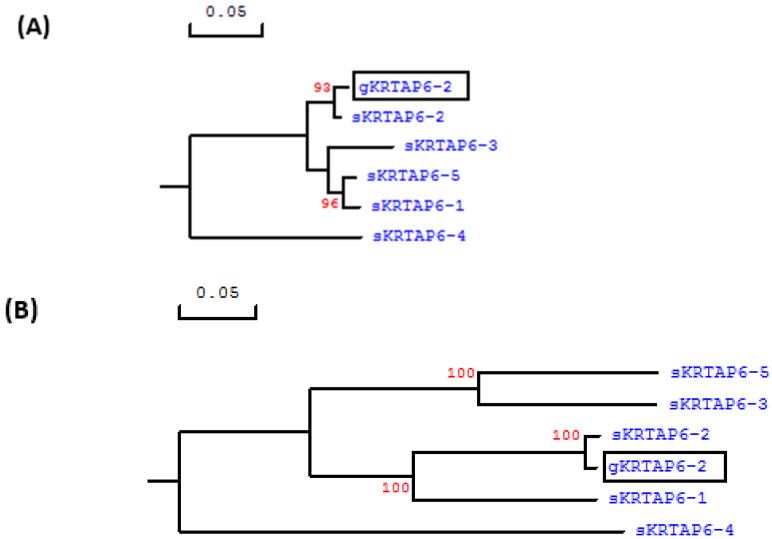
Phylogenetic trees constructed for the 300-bp sequences (**A**) upstream and (**B**) downstream the coding region of the putative goat *KRTAP6-2* and sheep *KRTAP6-n*. The goat and sheep *KRTAPs* are indicated with a prefix ‘g’ and ‘s’, respectively. The numbers at the forks show the bootstrap confidence value and only those ≥90% are shown. The scale bar indicates 0.05 nucleotide substitutions per site.

As the *KRTAP6-n* in sheep tend to share an elevated level of sequence similarity in the coding region, to confirm that this 252-bp ORF represented caprine *KRTAP6-2*, the 300-bp sequences upstream and downstream of this ORF in the Caprine Genome Assembly were also analyzed. A phylogenetic assessment confirmed that the upstream and downstream sequences of the ORF were more closely related to ovine *KRTAP6-2* than any other *KRTAP6-n* sequences from sheep (Figure 1). The location of this caprine ORF was found to be approximately 50 kb downstream of the *KRTAP20-2* sequence and approximately 89 kb upstream of *KRTAP20-1*. 

### 3.2. Identification of Nucleotide Sequence Variation in Caprine KRTAP6-2 

**Figure 2:**
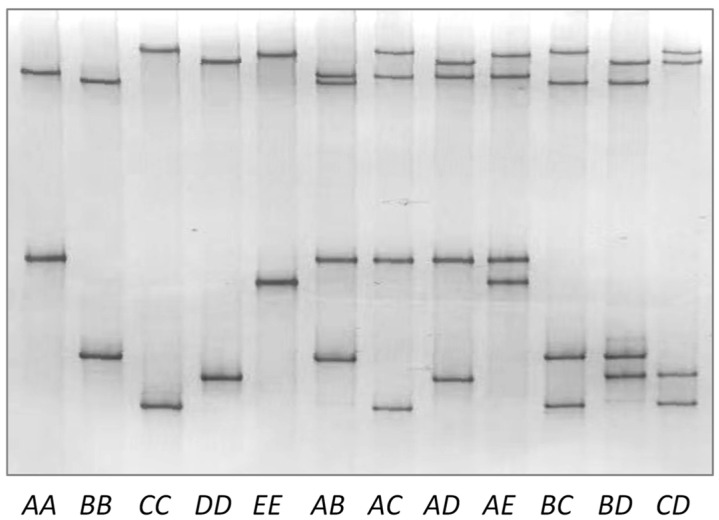
Polymerase chain reaction–single strand conformation polymorphism (PCR-SSCP) analysis of caprine *KRTAP6-2*. Five distinct banding patterns representing five different variants (*A*, *B*, *C*, *D*, and *E*) are shown in either homozygous or heterozygous forms. The entire gel image is shown in Figure S1.

In the 230 goats studied, different PCR-SSCP banding patterns were observed and these were resolved to suggest that there were five unique nucleotide sequences present in both homozygous and heterozygous genotypes (Figure 2). DNA sequencing of the PCR amplicons subsequently revealed five nucleotide sequences (named *A* to *E*; GenBank accession numbers OP157192-OP157196). All these sequences were different, but shared high sequence similarities (over 98%) to the goat *KRTAP6-2* sequence identified in the goat genome assembly sequence NC_030808.1. This suggests that these sequences were derived from the same *KRTAP6-2* locus and that the differences in the sequences may represent variation in the gene.

**Figure 3:**
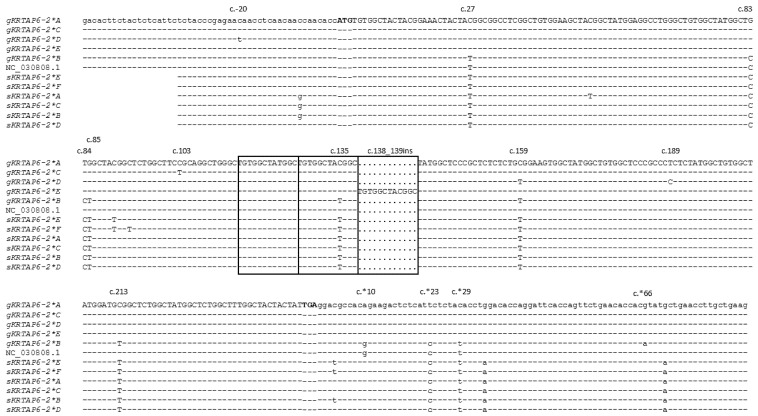
Sequence alignment of the goat *KRTAP6-2* variants identified in this study, together with the goat genome assembly sequence (NC_030808.1) and previously reported sheep variants. The goat sequences exclude the sequences of the primer binding regions and are labeled with the prefix ‘g’, whereas the sheep *KRTAP6-2* sequences are labeled with the prefix ‘s’. Nucleotides in the coding region are represent in upper case, whereas those in the non-coding region are in lower case. Nucleotide sequences identical to the top sequence are shown by dashes and dots represent the absence of nucleotides. The locations of the nucleotide sequence differences identified among the five caprine variants are indicated above the sequences. The ‘TGTGGCTA(T/C)GGC’ repeats are shown in boxes. The transcription start codon (ATG) and stop codon (TGA) are shown in bold. The sequences of ovine variants (*A*–*F*) are retrieved from GenBank with accession numbers KT725827–KT725832.

**Figure 4:**
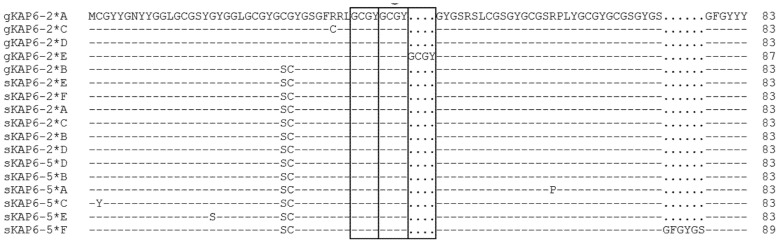
Alignment of the predicted amino acid sequences of the *KRTAP6-2* and *KRTAP6-5* variants identified in goats and sheep. Amino acids are represented in one letter code. Dashes represent amino acids identical to the top sequence and dots represent the absence of amino acids. The goat sequences are indicated with a prefix ‘g’, and the sheep sequences are with a prefix ‘s’. The GenBank accession numbers for sheep *KRTAP6-2* (*A*–*F*) are KT725827–KT725832, and those for sheep *KRTAP6-5* (*A*–*F*) are KT725841–KT725846. The GCGY repeats are shown in boxes.

Among the five caprine *KRTAP6-2* variants identified, there were 11 SNPs, a three-nucleotide sequence variation and an insertion/deletion of 12-bp sequence (Figure 3). Except for one of the SNPs (c.103C/T; p.Arg35Cys), all the other SNPs were either outside the coding region or did not lead to amino acid changes. The three-nucleotide sequence variation was located at c.83_c.85 and would result in two amino acid changes (p.Ser-Cys28_29Cys-Gly). The 12-bp insertion occurred in a tandem repeat of TGTGGCTA(T/C)GGC and led to variation in the number of repeats. There were three repeats in variant *E* and two repeats in all the other variants. This additional repeat unit in variant *E* would lead to the presence of an additional CGYG repeat in the central region of the protein (Figure 4). Variant *B* was notably different to all the other variants and possessed some unique nucleotide sequences that are observed in the ovine orthologue. In the coding region, variant *B* was identical to ovine variants *B*, *C*, and *D* (Figure 3). 

**Figure 5:**
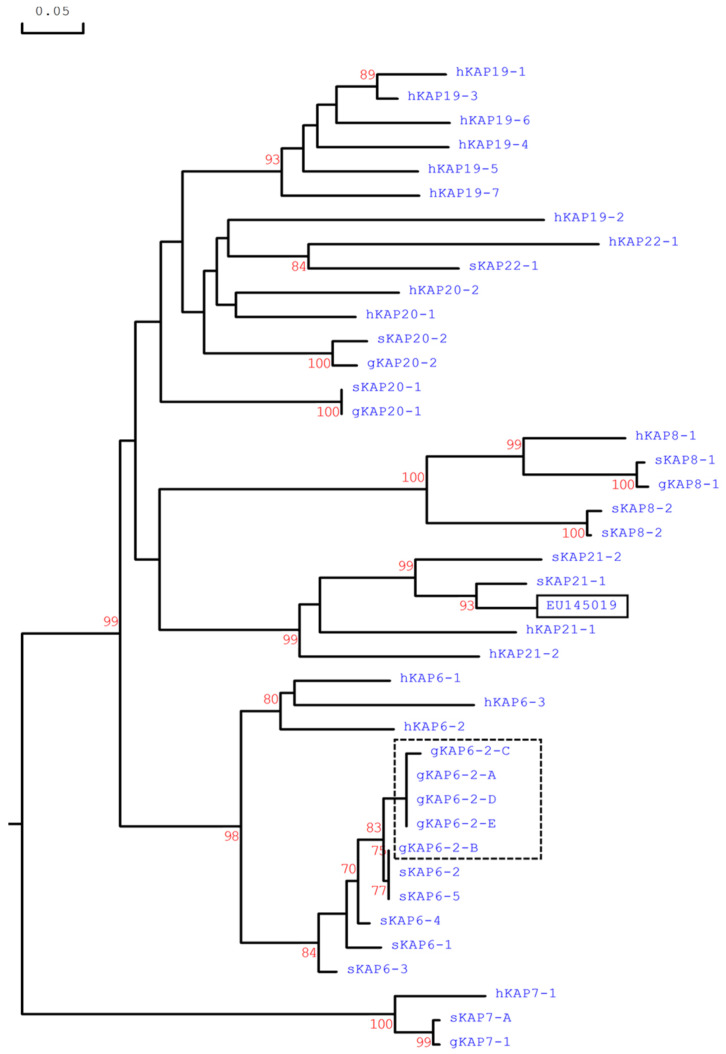
Phylogenetic tree of the newly identified caprine KAP6-2 variants, together with other HGT-KAPs identified in goats, sheep, and humans. The goat, sheep, and human KAPs are indicated with a prefix ‘g’, ‘s’, and ‘h’, respectively. The numbers at the forks show the bootstrap confidence value and only those ≥70% are shown. The scale bar indicates 0.05 amino acid substations per site. The caprine KAP6-2 variants are shown in a dashed box, and the GenBank accession numbers for other HGT-KAPs are: NM_001193399 (sKAP6-1), KT725827 (sKAP6-2), KT725833 (sKAP6-3), KT725838 (sKAP6-4), KT725841 (sKAP6-5), AY510121 (gKAP7-1), X05638 (sKAP7-1), NM_181606 (hKAP7-1), AY510122 (gKAP8-1), X05639 (sKAP8-1), NM_175857 (hKAP8-1), AY510123 (gKAP8-2), KF220646 (sKAP8-2), NM_181607 (hKAP19-1), NM_181608 (hKAP19-2), NM_181609 (hKAP19-3), NM_181610 (hKAP19-4), NM_181611 (hKAP19-5), NM_00130312 (hKAP19-6), NM_181614 (hKAP19-7), MG742218 (gKAP20-1), MF973462 (gKAP20-2), MH243552 (sKAP20-1), MH071391 (sKAP20-2), NM_181615 (hKAP20-1), NM_181616 (hKAP20-2), MF143980 (sKAP21-1), MF143975 (sKAP21-2), NM_181619 (hKAP21-1), NM_181617 (hKAP21-2), KX377616 (sKAP22-1), and NM_181620 (hKAP22-1). The goat sequence EU145019 that was previously reported as ‘KAP6-2’ is shown in a box.

Phylogenetic analysis of the translated amino acid sequences revealed that the caprine *KRTAP6-2* sequences identified here were closer to ovine *KRTAP6-2* and ovine *KRTAP6-5*, but different to other known HGT-*KRTAP* sequences (Figure 5). In contrast, the previously reported “caprine *KRTAP6-2*” sequence (EU145019) was separate to all the clustered *KRTAP6-n* sequences, but instead clustered with *KRTAP21-n*, supporting the contention that the sequence of EU145010 is not caprine *KRTAP6-2*. 

The caprine *KRTAP6-2* sequences would encode polypeptide with 83 and 87 amino acids, respectively. The polypeptides would contain a high content of glycine (38.6–40.2 mol %) and tyrosine (21.7–21.8 mol %) and a moderate level of cysteine (12.0–12.6 mol %) and serine (9.6–10.8 mol %). The caprine KAP6-2 proteins are predicted to be basic proteins with isoelectric point (pI) of 8.27–8.51.

### 3.3. Association of KRTAP6-2 Variation with Cashmere Fiber Traits 

In the 230 goats investigated, 11 genotypes were detected, and these were: *AA* (*n* = 54), *AB* (*n* = 67), *AC* (*n* = 15), *AD* (*n* = 2.7), *AE* (*n* = 5), *BB* (*n* = 15), *BC* (*n* = 11), *BD* (*n* = 21), *CC* (*n* = 5), *CD* (*n* = 6), *DD* (*n* = 3), and *EE* (*n* = 1). This gives variant frequencies of 48.3%, 28.0%, 9.1%, 13.0%, and 1.5% for *A* to *E*, respectively. 

As variant *E* was found in only six goats (with a frequency of less than 5%), these six goats were removed from the association study and the effect of variant *E* on cashmere fiber traits was not analyzed given the potential for these goats to bias the analyses. In the presence/absence models, the presence of variant *D* was found to be associated with decreased MFD (present: 13.26 ± 0.07 µm; absent: 13.55 ± 0.04 µm; *p* < 0.001) (Table 1). No association was revealed with crimped fiber length or cashmere yield. 

## 4. Discussion

This study describes the identification of a new caprine *KRTAP*, sequence variation in the gene and an association between this variation and cashmere MFD in Longdong cashmere goats. The chromosomal location of this newly identified *KRTAP* sequence matches well with the location of *KRTAP6-2* reported in sheep [6], and the flanking sequences of this *KRTAP* were more closely related to ovine *KRTAP6-2* flanking regions than any other ovine *KRTAP6-n*, suggesting that the newly identified *KRTAP* represent caprine *KRTAP6-2*. The caprine *KRTAP6-2* is predicted to encode a basic protein that contains over 60 mol % of glycine and tyrosine, and this is consistent with the characteristics of an HGT-KAP and the observation that in sheep all known HGT-KAPs are basic proteins, except for KAP8-2 [16].

While the number of variants and the nature of SNPs detected for caprine *KRTAP6-2* is comparable to that reported for its sheep orthologue [17], some unique features are observed for caprine *KRTAP6-2*. First is the high density of SNPs. The identification of 11 SNPs in the amplified region of 389-bp (excluding the primer binding regions) gives a density of 28.2 SNPs per kb. While *KRTAPs* tend to have high density of SNPs [6], the SNP density observed is much higher than its ovine orthologue, and higher than all the *KRTAPs* identified in sheep except *KRTAP1-3*.

The presence of a three-nucleotide sequence variation in the coding sequence of caprine *KRTAP6-2* is also notable. Despite this kind of variation (called trinucleotide polymorphisms; TNPs) being frequently found in the human genome, they are almost completely absent from coding exons and there are only three coding TNPs described in the Chinese human genome sequence and six from the Venter genome sequence [25]. In humans, coding TNPs are reported for *KRTAP10-1* [25], but these TNPs only lead to one amino acid change, whereas the TNP described here in caprine *KRTAP6-2* would lead to a change of two amino acids. The functional effect of the goat *KRTAP6-2* TNP awaits further investigation, but the lack of association of variant *B* with cashmere traits (Table 1) suggests that this TNP is unlikely to have an effect on fiber diameter and cashmere weight.

There is also length variation in the novel *KRTAP6-2* sequences. Given that all the variants were the same length except for variant *E* which had an extra 12-bp in the tandem repeat region, it is likely that the length variation detected is due to the insertion of the 12-bp repeat in variant *E*, rather than the deletion of the 12-bp repeat in all the other caprine *KRTAP6-2* variants. In sheep, length variation has been described for *KRTAP6-1*, *KRTAP6-3*, and *KRTAP6-5*, but it has not been detected for *KRTAP6-2* and *KRTAP6-4* [17,26].

The variant *B* sequence appears to have diverged from the other caprine variants to be more like the ovine *KRTAP6-2* variants, most notably being identical to some of the ovine variants in the coding region (Figure 3). This phenomenon has not been reported for any other *KRTAPs*, and its genesis is therefore uncertain. Given that the sequence variation reported here was found in only 230 Longdong goats from a single farm, more variants and additional variation may be found when more goats from different farms are investigated. This is supported by the differences observed between the sequences reported here and the goat genome assembly sequence NC_030808.1.

Caprine *KRTAP6-2* is clustered with *KRTAP20-1* and *KRTAP20-2* and located between them on chromosome 1, but the association detected *KRTAP6-2* variation and MFD is different to that reported for the nearby genes. Previous research with Longdong goats has revealed that variation in *KRTAP20-1* and *KRTAP20-2* are associated with cashmere fiber weight and crimped fiber length, but not MFD [1,12]. This suggests that the MFD association detected for *KRTAP6-2* may be because of *KRTAP6-2* itself and not because of *KRTAP20-1* and *KRTAP20-2*, but in sheep, there are additional *KRTAPs* clustered with *KRTAP6-2* in the region between *KRTAP20-1* and *KRTAP20-2*—including *KRTAP6-1*, *KRTAP6-3*, *KRTAP6-4*, *KRTAP6-5*, and *KRTAP22-1* [6]. Further investigation of these genes in goats and whether they associate with cashmere fiber traits may provide more information on the role of *KRTAP6-2* in fiber traits and the other genes too.

The detection of association between variant *D* and cashmere MFD suggests variation in caprine affects the trait. Given that the association was only detected for variant *D*, but not *A*, and that these two variants differed by a single SNP (c.-20C/T) in the 5′-UTR and two synonymous SNPs (c.159C/T and c.189T/C) in the coding region, the effect detected for variant *D* may be due to some of or all these SNPs. Despite not causing amino acid sequence changes, SNPs within the 5′-UTR, or synonymous SNPs in coding regions may nevertheless affect mRNA translation efficiency [27].

The MFD of cashmere is an economically important trait. The associations found in this study suggest that caprine *KRTAP6-2* may have potential as a gene-marker for selection for cashmere fiber traits that are of greater value in cashmere production.

## 5. Conclusions

This study identified the KAP6-2 gene in goats and revealed various types of sequence variation present in the gene, including SNPs, a TNP, and an insertion/deletion. Variation in caprine *KRTAP6-2* was found to be associated with variation in MFD, suggesting that the gene may have value in the development of gene markers for improving a key cashmere fiber trait.

## Figures and Tables

**Table 1:** Association of caprine *KRTAP6-2* variants with cashmere fiber traits.

Trait	Variant	Absent		Present		*p* ^2^
		Mean ± SE ^1^	*n*	Mean ± SE	*n*	
**Cashmere yield (g)**	*A*	411.87 ± 8.34	61	412.62 ± 4.75	163	0.941
	*B*	412.86 ± 5.02	110	411.97 ± 5.09	114	0.889
	*C*	412.33 ± 4.15	187	412.84 ± 7.72	37	0.951
	*D*	412.97 ± 4.29	167	410.49 ± 7.28	57	0.753
**Mean fiber diameter (µm)**	*A*	13.51 ± 0.08	61	13.47 ± 0.05	163	0.751
	*B*	13.47 ± 0.05	110	13.50 ± 0.05	114	0.651
	*C*	13.47 ± 0.04	187	13.54 ± 0.07	37	0.390
	*D*	13.55 ± 0.04	167	13.26 ± 0.07	57	<**0.001**
**Crimped fiber length (cm)**	*A*	4.19 ± 0.09	61	4.24 ± 0.05	163	0.631
	*B*	4.27 ± 0.05	110	4.19 ± 0.06	114	0.213
	*C*	4.24 ± 0.05	187	4.18 ± 0.08	37	0.517
	*D*	4.21 ± 0.05	167	4.29 ± 0.08	57	0.369

^1^ Estimated marginal means and standard errors derived from general linear mixed-effects models that included ‘gender’ as a fixed factor, and ‘sire’ as a random factor. ^2^ *p* < 0.05 are in bold.

## Data Availability

The original data used in this paper are available by contacting the corresponding author upon request.

## Supplementary Materials

The following supporting information can be downloaded at: https://www.mdpi.com/article/10.3390/ani12162040/s1, Figure S1: The original picture of the entire gel.
