# Supplementary figures and images for: Sequence Variation in Caprine KRTAP6-2 Affects Cashmere Fiber Diameter
Source: Animals (Basel). 2022 Aug 11;12(16):2040. doi: 10.3390/ani12162040 (PMC9404442; doi:10.3390/ani12162040)

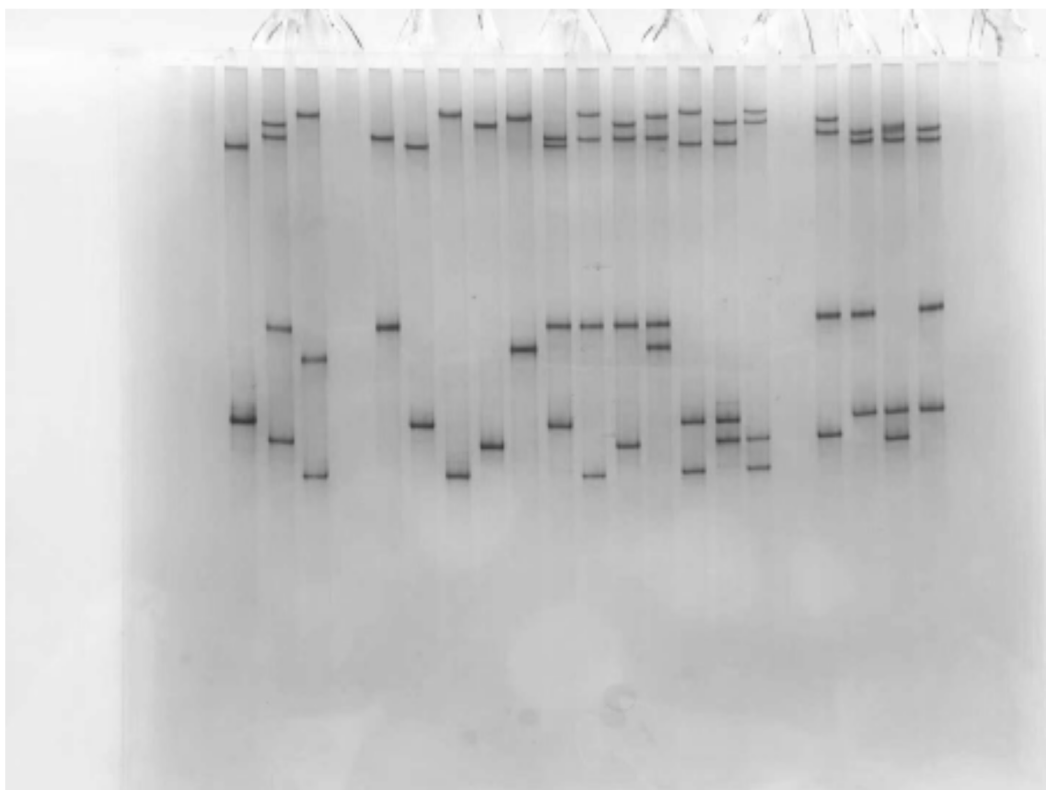

**Figure S1.** The original picture of the entire gel.

Supplement: Supplementary file 1 [file animals-12-02040-s001.zip › animals-1821362-Supplementary.pdf]
